# A comparative molecular and 3-dimensional structural investigation into cross-continental and novel avian *Trypanosoma* spp. in Australia

**DOI:** 10.1186/s13071-017-2173-x

**Published:** 2017-05-12

**Authors:** Crystal Cooper, R. C. Andrew Thompson, Adriana Botero, Amanda Kristancic, Christopher Peacock, Yaowanuj Kirilak, Peta L. Clode

**Affiliations:** 1 Centre for Microscopy, Characterisation and Analysis, The University of Western Australia, Crawley, WA 6009 Australia; 2 School of Veterinary and Life Sciences, Murdoch University, Murdoch, WA 6150 Australia; 3 Marshall Centre, School of Pathology and Laboratory and Medical Sciences, University of Western Australia, Crawley, WA 6009 Australia; 4 Telethon Kids Institute, 100 Roberts Rd, Subiaco, WA 6008 Australia

**Keywords:** *Trypanosoma*, Australian birds, 18S rDNA, FIB-SEM, Mitochondrion, Kinetoplast

## Abstract

**Background:**

Molecular and structural information on avian *Trypanosoma* spp. throughout Australia is limited despite their intrinsic value in understanding trypanosomatid evolution, diversity, and structural biology. In Western Australia tissue samples (*n* = 429) extracted from 93 birds in 25 bird species were screened using generic PCR primers to investigate the diversity of *Trypanosoma* spp. To investigate avian trypanosome structural biology the first 3-dimensional ultrastructural models of a *Trypanosoma* spp. (*Trypanosoma* sp. AAT) isolated from a bird (currawong, *Strepera* spp.) were generated using focussed ion beam milling combined with scanning electron microscopy (FIB-SEM).

**Results:**

Here, we confirm four intercontinental species of avian trypanosomes in native Australian birds, and identify a new avian *Trypanosoma*. Trypanosome infection was identified in 18 birds from 13 different bird species (19%). A single new genotype was isolated and found to be closely related to *T. culicavium* (*Trypanosoma* sp. CC2016 B002). Other *Trypanosoma* spp. identified include *T. avium*, *T. culicavium*, *T. thomasbancrofti*, *Trypanosoma* sp. TL.AQ.22, *Trypanosoma* sp. AAT, and an uncharacterised *Trypanosoma* sp. (group C-III *sensu* Zidková et al. (Infect Genet Evol 12:102-112, 2012)), all previously identified in Australia or other continents. Serially-sectioning *Trypanosoma* sp. AAT epimastigotes using FIB-SEM revealed the disc-shaped kinetoplast pocket attached perpendicular to the branching mitochondrion. Additionally, the universal minicircle sequence within the kinetoplast DNA and the associated binding protein were determined in *Trypanosoma* sp. AAT.

**Conclusions:**

These results indicate that bird trypanosomes are relatively conserved across continents, while being locally diverse, which supports the hypothesis that bird trypanosomes exist as fewer species than described in the literature. Evidence exists that avian *Trypanosoma* spp. are infecting mammals and could be transmitted by haemadipsid leeches. *Trypanosoma* sp. AAT is most likely a separate species currently found only in Australia and the first 3-dimentional ultrastructural analysis of an avian trypanosome provides interesting information on their morphology and organelle arrangement.

**Electronic supplementary material:**

The online version of this article (doi:10.1186/s13071-017-2173-x) contains supplementary material, which is available to authorized users.

## Background

Parasites from the genus *Trypanosoma* are protozoan flagellates that infect almost all known animal taxa and are responsible for a number of neglected diseases in humans and livestock. The biodiversity and biology of trypanosomes infecting wildlife worldwide is still an area that is poorly understood, especially in birds. The prevalence of bird trypanosomes is difficult to assess accurately because of low parasitaemia, morphological tropism in the host, and development in the bone marrow rather than peripheral blood [1–3]. In the past, avian trypanosomes were often presumed to be species-specific, which resulted in the description of almost 100 species. As early as 1974 researchers proposed the actual number of species present in birds was likely to be much lower and that the taxonomic descriptions of avian trypanosomes should be revised [4–6]. Researchers suggested that investigating blood smears taken from birds to identify trypanosomes was outlasting its usefulness, despite being the most popular method of screening for trypanosome infection [5]. Recent phylogenetic analyses of bird trypanosomes indicate there are less bird trypanosomes than currently described and the majority most likely belong to one of three trypanosome species (*Trypanosoma corvi*, *Trypanosoma avium* or *Trypanosoma bennetti*) with the total number of species closer to 12 [7, 8]. Despite this reduction in the number of avian *Trypanosoma* spp. they are still a polyphyletic group [8].

Investigations into the trypanosomes of Australian wildlife in the past have demonstrated high levels of genetic and morphological diversity, mixed infections, and a lack of species specificity [9]. In Australia, trypanosomes have been described from a variety of wild birds [10] and while there are six *Trypanosoma* spp. that have been described in Australian birds since 1910 [10–13] there is a need for taxonomic redescription to ensure current described species are confirmed/correlated with molecular sequence data [6, 9]. *Trypanosoma* sp. AAT was the first trypanosome isolated in vitro from an Australian bird, the currawong (*Strepera* sp.) that was also characterised using molecular techniques [14]. *Trypanosoma* sp. AAT has been utilised in a number of genetic investigations representing an Australian trypanosome [14, 15]. The morphological information provided on *Trypanosoma* sp. AAT were two images of epimastigotes exhibiting long flagella and molecular sequences from the small ribosomal subunit 18S (18S rDNA) and glyceraldehyde 3-phosphate dehydrogenase (*GAPDH*) gene regions. A subsequent study in Australia that described *Trypanosoma thomasbancrofti* from the regent honeyeater (*Anthochaera phrygia*) and isolated *T. avium* from noisy minor birds (*Manorina melanocephala*) also provided sequences from the 18S rDNA and *GAPDH* gene regions [13].

The class Kinetoplastida, which contains all trypanosomes has a unique organelle called the kinetoplast containing extra-nuclear DNA (kDNA). The kDNA is contained inside the kinetoplast pocket that is part of the single large mitochondrion [16, 17], which is unusual in both its structural and functional properties [18, 19]. The structure and size of *Trypanosoma* mitochondria are variable and cell cycle dependant. This may be due to available carbon sources used in metabolism, especially high levels of glucose as this facilitates the transition from oxidative phosphorylation to glycolysis and reduces the size of the mitochondrion [20–22]. Additionally, kinetoplastids have a higher percentage of their DNA in their mitochondrion compared to other eukaryotes [16]. For example, *Trypanosoma cruzi* the causative agent of Chagas disease can have up to 30% of its DNA in the kinetoplast-mitochondrion [23]. The kDNA is made up of circular maxicircles and minicircles that appear as a network inside the kinetoplast pocket, which is attached to the flagellum by the tripartite attachment complex (TAC) [24]. Maxicircles that encode for mRNA’s require RNA editing (post-transcriptional process of uridine insertion or deletion), which is achieved through guide RNA’s that are encoded on the minicircles [25]. Minicircle size and kDNA width have been used in the past to differentiate between trypanosomes isolated from birds [26]. The kDNA minicircles contain the ‘universal minicircle sequence’ (UMS or CSB-3), a 12- mer sequence (5'-GGGGTTGGTGTA-3'), which is conserved in most trypanosomatids although, the location and number of times it is present within the minicircles differ between species [27]. In addition to this there are two other conserved sequence blocks within the minicircles (CSB-1, CSB-2). The UMS is the specific binding site for the UMS-binding protein (UMSBP), which is involved in kDNA replication [28] and anti UMSBP from *Crithidia fasciculata* has been used to detect homologous proteins in other trypanosomatids such as *T. cruzi*, *Leishmania donovani* and *T. brucei* [28–30]. Some trypanosomes isolated from birds such as *T. avium* possess what appears to be a unique kinetoplast, with unusually large minicircles [26, 31, 32] that make them intrinsically important tool in trying to understand kinetoplastid evolution and biology. As a result of the pleomorphism of avian trypanosomes within species, it has been suggested that in vitro investigations may be more reliable when trying to characterise their structure and understand the differences between species as in vitro studies provide a more stable environment [6, 33].

When investigating trypanosomes using current microscopy techniques there are a number of difficulties associated with interpreting 2-dimensional images in relation to the 3-dimensional (3D) organism. This is especially apparent in transmission electron microscopy (TEM), which allows for high resolution ultrastructural analysis in cells, but the sample sections only represent a small ~100 nm-thick segment of the cell. With this, the angle the section has been cut through a structure is usually not known making feature identification and accurate quantifiable measurements, at times difficult. The recent improvement in automated, high-throughput imaging techniques capable of high resolution 3D imaging overcomes these issues, and has led to 3D cellular architecture being characterised in *Trypanosoma brucei* [17, 34–36], *T. cruzi* [37, 38] and *T. dionisii* [39]. A variety of morphological forms have been studied using a variety of different techniques that are available, including super resolution confocal microscopy [17], electron tomography [34, 37], serial block-face scanning electron microscopy [17, 36, 40], and focussed ion beam milling combined with scanning electron microscopy (FIB-SEM) [34, 38]. The primary difference between the latter two SEM-based techniques is that the serial block face method uses a knife to cut through the sample slice by slice permitting analysis areas in the order of hundreds of microns, while the FIB-SEM uses a beam of ions to cut through the sample permitting analyses on the scale of tens of microns.

The trypanosomes infecting birds must be better studied in order to understand trypanosome evolution, diversity, and structural biology. Therefore, the aim of this study was to resolve the taxonomy of Australian bird trypanosomes using molecular techniques, including, for the first time, a number of bird species from Western Australia. Additionally, an ultrastructural analysis of *Trypanosoma* sp. AAT was undertaken in order to better understand structural characteristics of an Australian avian trypanosome.

## Methods

### Collection of birds, 18S rDNA amplification, and sequencing of bird tissues

Specimens were collected opportunistically from dead birds handed into Murdoch University between 2008 and 2014 (sources included roadkill and fauna rehabilitation hospitals). Thirty Australian ravens (*Corvus coronoides*) and 13 Australian magpies (*Cracticus tibicen*) were included in the study along with a number of bird species residing in Australia (Additional file 1: Table S1). Birds were frozen at -20 °C following collection and dissected between March and December 2015. Dissected tissues were stored in 70% ethanol taking precautions to prevent cross-contamination between tissues. Up to seven tissues were extracted from 49 birds (*n* = 334) and up to three tissues extracted from 44 birds (*n* = 95) (Additional file 1). Genomic DNA was extracted using Quiagen Blood and Tissue Mini-kit (Quigen, Hilden, Germany) following the manufacturer’s protocols for tissue extraction including both positive and negative controls. All amplification reactions were performed in a PT100 thermocycler (MJ-Research). Trypanosome DNA was amplified using generic 18S rDNA PCR primers (SLF, S762R, S823F and S662R) in a nested PCR protocol [41, 42], under the following conditions: denaturation step at 94 °C for 5 min, followed by 35 cycles of 30 s at 94 °C, 30 s at 52 °C, 50 s at 72 °C, and a final extension step at 72 °C for 7 min. Both external and internal reactions contained 25 μl consisting of 1 μl of DNA, 0.8 μM of each primer, 0.2 mM of dNTPs, 2 mM of MgCl_2_ and 0.2 μl of Taq DNA polymerase for the external reaction. The internal reaction contained 1 μl of DNA, 0.8 μM of each primer, 0.2 mM of dNTPs, 1.5 mM of MgCl_2_ and 0.2 μl of Taq DNA polymerase. Due to the amplification of bird DNA by these primers tissues were also screened for trypanosomes using a subsequent nested primer set described in Noyes et al. [43] including external (TRY927F, TRY927R), and internal (SSU561F, SSU561R) primers under the following conditions: denaturation step at 94 °C for 5 min, followed by 30 cycles of 30 s at 94 °C, 1 min at 55 °C, 1 min and 30 s at 72 °C, and a final extension step at 72 °C for 10 min. Both external and internal reactions contained 25 μl consisting of 2 μl of DNA, 0.2 μM of each primer, 0.32 mM of dNTPs, 3 mM of MgCl_2_ and 0.2 μl of Taq DNA polymerase. PCR products were purified by cutting DNA bands using gel cutting tips from 2% agarose gel and using the freeze-squeeze method [44]. Samples were sequenced in both directions using an ABI Prism™ Terminator Cycle Sequencing Kit on an Applied Bio-system 3730 DNA Analyser (Applied Bio-systems, California, USA). Sequences were edited and aligned using Geneious 8.1 [45].

### Phylogenetic analysis

A multiple-sequence alignment containing the 26 sequences isolated in this study from Western Australian birds, 47 sequences from trypanosome isolates downloaded from GenBank including two outgroups (*T. brucei*) was conducted using the MUSCLE [46] plugin for Geneious v. 8.1 [45]. A GTR + G + I substitution model selected by jModelTest [47] was used in the construction of maximum-likelihood, neighbour-joining, and Bayesian analysis trees. Bootstrap support for 1000 replicates was performed for maximum-likelihood and neighbour-joining tree searches using MEGA 6 [48]. A Bayesian analysis (10,000,000 generations, sampling frequency of 1000, ‘burn-in’ 3000) was run in Mr Bayes v. 3.1.2 [49] plugin for Geneious v. 8.1. The final tree was edited using Inkscape.

### Maintenance of trypanosomes in culture

Two vials of *Trypanosoma* sp. AAT originally isolated in 2004 [14] were donated by Dr J. R. Stevens from Bristol University, UK and were identified using generic trypanosome primers described in section 2.1 [41, 42]. Trypanosomes were grown in an incubator at 28 °C with 5% CO_2_. Cultures were maintained in biphasic medium containing Brain-heart infusion (BHI), BBL agar- grade A, 0.48% gentamicin, and 10% defibrinated rabbit blood as a solid phase, and either RPMI 1640 (Roswell Park Memorial Institute 1640) supplemented with tryptose (TRPMI) as in Noyes et al. [43], or Grace’s insect media [50] as a liquid phase. All liquid media was supplemented with 10% heat-inactivated foetal calf serum (FCS) and 1% penicillin-streptomycin.

### Scanning and transmission electron microscopy

For scanning electron microscopy (SEM), trypanosomes were fixed in 2.5% glutaraldehyde in 1× PBS and stored at 4 °C before being mounted on poly-L-lysine coated coverslips, dehydrated through a series of ethanol solutions (30%, 50%, 70%, 90%, 100%, 100%) using a PELCO Biowave then processed in a critical point drier. Coverslips were mounted on stubs with adhesive carbon, coated with 2 nm platinum (Pt) and 10 nm carbon. Trypanosomes were imaged at 3 kV using the in-lens secondary electron detector on a Zeiss 55VP field emission SEM. For transmission electron microscopy (TEM) trypanosomes were fixed in 2.5% glutaraldehyde in 1× PBS and processed using a PELCO Biowave microwave, where samples were post-fixed in 1% OsO_4_ in 1× PBS followed by progressive dehydration in ethanol (30%, 50%, 70%, 90%, 100%) then acetone, before being infiltrated and embedded overnight at 70 °C in epoxy resin Procure-Araldite. Sections ~120 nm-thick were cut with a diamond knife on a Leica microtome and mounted on copper grids. Digital images were collected from unstained sections at 120 kV on a JEOL 2100 TEM fitted with a Gatan ORIUS1000 camera.

### Focused ion beam - scanning electron microscopy

*Trypanosoma* sp. AAT samples were fixed in 2.5% glutaraldehyde in 1× PBS and stored at 4 °C before heavy metal staining, which had been adapted from previous methods [40, 51]. Samples were washed in 1× PBS three times then processed through 2% osmium tetroxide + 1.5% potassium ferricyanide for 1 h, thiocarbohydrazide for 20 min, 2% osmium tetroxide for 30 min, and finally left in 1% aqueous uranyl acetate overnight at 4 °C. The following day samples were stained with lead aspartate (20 mM lead nitrate in 0.03 M L-aspartic acid adjusted to pH 5.5 with 1 M KOH) for 1 h [40]. Samples were washed with water thoroughly between each stain three times for a duration of 5 min. Following staining, samples were dehydrated through a series of ethanol (20%, 50%, 70%, 90%, 100%, 100%) followed by acetone, and infiltrated using Hard plus resin 812 (25%, 50%, 75%, 100%) [51]. Samples were embedded in fresh 100% resin before being cured at 70 °C for 48 h. Samples were trimmed using a glass knife on a Leica microtome to locate sites of interest, attached to a 45 degree-angled metal stub using aluminium tape, coated with 10 nm Pt, and prepared and imaged in a FEI Helios G3 FIB-SEM. To prepare sites of interest, a protective coating of 1 μm Pt was deposited on the top surface of the region of interest (ROI) and side trenches (~15 μm^3^) were dug either side. The face of the ROI was then progressively milled and imaged in 25 nm steps. Milling was performed at 7 degrees (=52 degree actual tilt) and a working distance of ~4 mm using a Ga beam at 0.79 nA and 30 kV. Back scattered electron images were collected after each milling step at 45 degrees (=0 degree actual tilt) and a 2 mm working distance using a through lens electron detector (TLD) at 2 kV and 0.69 nA beam current. 3D data were aligned and processed using Amira 6.0 for FEI systems.

### UMS amplification and sequencing

A fragment of *Trypanosoma* sp. AAT minicircles was amplified by PCR using the UMS as a forward primer (UMSF 5'-GGG GTT GGT GTA-3'), and its complementary sequence as a reverse primer (UMSR 5'-TAC ACC AAC CCC-3') under the following conditions: an initial step of 95 °C for 5 min, followed by 35 cycles of 30 s at 95 °C, 30 s at 40 °C, and 60 s at 72 °C, and a final extension step of 5 min at 72 °C. Reactions contained 25 μl consisting of 1 μl of DNA, 1.5 mM of each primer, 200 mM of dNTPs, 0.2 μl of Taq DNA polymerase, and 1.5 mM of MgCl_2_. DNA was purified using an Agencourt AMPure PCR Purification system (manufacturer’s instructions). Purified amplicons were sequenced using an ABI Prism™ Terminator Cycle Sequencing kit (Applied Bio-systems, California, Foster City, USA) on an Applied Bio-system 3730 DNA Analyzer. Sequences were aligned with the minicircles of *T. rangeli* (accession number L19395T), *T. lewisi* (accession number M17995), two strains of *T. cruzi* (accession numbers X04680, M18814), and two strains of *T. copemani* (accession numbers TBA) using MUSCLE [46].

### Western blot

Total protein extract from *Trypanosoma* sp. AAT epimastigotes was obtained by centrifugation of 1 × 10^6^ epimastigotes at 8000 × *g* for 5 min. The pellet was washed, centrifuged twice in 1× PBS, and resuspended in 40 μl of water. Subsequently, 10 μl of 10% SDS (Sodium Dodecyl Sulfate) was added and samples were sonicated for 5 min. After sonication, cell lysates were solubilised in cracking buffer containing final concentrations of 50 mM Tris-HCl, pH 6.8, 4% SDS (wt/vol), 3.5% (vol/vol) beta-mercaptoethanol, 10% (vol/vol) glycerol, and 10 mM EDTA. The solution was centrifuged at 10,000 × *g* for 30 min at 4 °C. The protein concentration was determined using the direct-detect assay free-cards (EMD millipore corporation, Billerica, USA). Protein extract (30 μg) was boiled at 70 °C for 10 min and loaded onto a NuPag10 4-12% Bis-Tris gel. Protein bands on the gel were transferred onto a nitrocellulose membrane using the turbo-transfer system (Biorad, Hercules, USA). The membrane was blocked by incubation in 5% skim dry milk -diluted in TBST (tris-buffer solution tween) for 2 h with constant shaking, and probed with a 1:4000 dilution of *Crithidia fasciculata* anti-UMSBP overnight at 4 °C. The membrane was then washed three times with TBST and then incubated with a 1:10,000 dilution of ECL peroxidase labelled conjugated anti-rabbit secondary antibody (Jackson ImmunoResearch Laboratories, Inc. West Grove, USA) for 2 h followed by ECL detection as recommended by the manufacturer (Amersham Pharmacia Biotech, Piscataway, USA).

## Results

### Bird tissues screened for *Trypanosoma* spp.

**Fig. 1:**
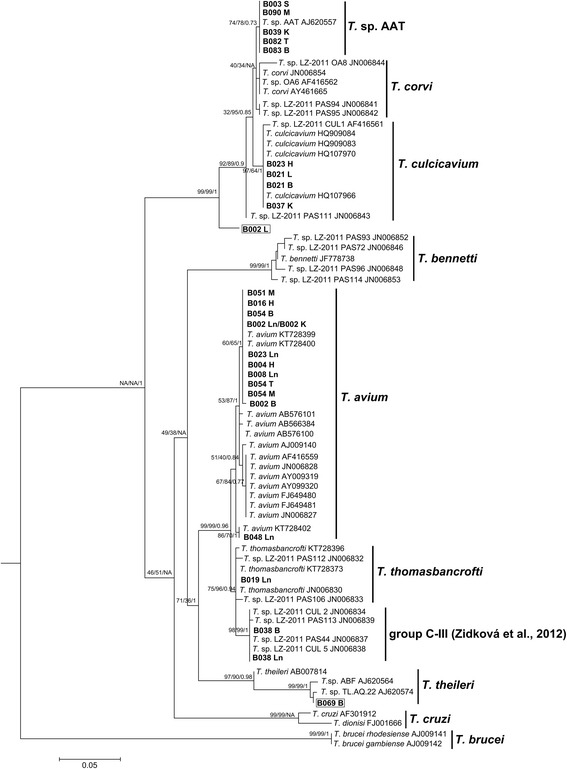
Phylogenetic analysis of avian *Trypanosoma* spp. based on partial ribosomal subunit (18S rDNA) sequences. This tree was generated using maximum likelihood with a GTR + G + I DNA substitution model. Numbers adjacent to isolate names indicate GenBank accession numbers and sequences acquired in this study are in bold, which represent the bird identification code. The samples in boxes were novel isolates. The first two numbers at the nodes indicate bootstrap support for 1000 searches for maximum-likelihood and neighbour-joining, respectively. The final number indicates Bayesian posterior probability. *Trypanosoma brucei rhodesiense* and *Trypanosoma brucei gambiense* are used as outgroups. Scale-bar indicates substitution per site

Tissue samples (*n* = 429) extracted from 93 dead birds across 25 species (Additional file 1: Table S1) were screened for trypanosome infection by PCR and 19% (*n* = 18) of birds, comprising 13 different species, were infected with trypanosomes from seven different species groups (Table 1). One novel genotype (*Trypanosoma* sp. CC2016 B002) was identified from the boobook (*Ninox novaeseelandiae*) (that was also infected with *T. avium*), which was submitted to GenBank (accession number KY425593). Of the seven avian trypanosomes identified, four have a cross-continental distribution. One isolate has only been found in Australia, *Trypanosoma* sp. AAT. The remaining isolate *Trypanosoma* sp. CC2016 B069 was most closely related to an undescribed trypanosome in the *T. theileri* clade (*Trypanosoma* sp. TL.AQ.22) found in a terrestrial leech (Haemadipsidae) in Australia [14] and was submitted to GenBank (accession number KY425594). Mixed infections were observed in a boobook (*Ninox novaeseelandiae*) and an Australian magpie (*Cracticus tibicen*), and four different species of *Trypanosoma* were identified from different Australian magpies. A phylogenetic analysis of trypanosome isolates demonstrated the length of partial 18S rDNA amplified (506 bp located towards the middle of the gene region) was sufficient to separate all isolates to the species level (Fig. 1).

**Table 1:** Bird tissues that tested positive for *Trypanosoma* spp. Bird identification number (ID), bird species, tissue, and *Trypanosoma* spp. are included. Tissues extracted include striated or skeletal muscle (Sm), heart (H), liver (L), spleen (S), lung (Ln), thoracic muscle (T) and brain (B)

ID	Bird species	Tissue	*Trypanosoma* spp.
B002	Boobook (*Ninox novaeseelandiae*)	L	*Trypanosoma* sp. CC2016 B002
		Ln, B, K	*T. avium*
B003	Currawong^a^ (*Strepera* spp*.*)	Sm	*Trypanosoma* sp. AAT
B004	Falcon (*Falco* spp*.*)	H	*T. avium*
B008	Barn owl (*Tyto alba*)	Ln	*T. avium*
B016	Australian dove^a^ (Family Columbidae)	H	*T. avium*
B019	Australian magpie^a^ (*Cracticus tibicen*)	Ln	*T. thomasbancrofti*
B021	Singing honeyeater^a^ (*Gavicalis virescens*)	B, L	*T. culicavium*
B023	Australian magpie (*Cracticus tibicen*)	Ln	*T. avium*
		H	*T. culicavium*
B037	Silvereye (*Zosterops lateralis*)	K	*T. culicavium*
B038	Australian mudlark (*Grallina cyanoleuca*)	Ln, B	*Trypanosoma* sp. C-111 [8]
B039	Pink and grey galah (*Eolophus roseicapilla*)	K	*Trypanosoma* sp. AAT
B048	Pigeon (*Columba livia*)	Ln	*T. avium*
B051	Australian raven (*Corvus coronoides*)	Sm	*T. avium*
B054	Australian raven (*Corvus coronoides*)	Sm, B, T	*T. avium*
B069	Australian raven (*Corvus coronoides*)	B	*Trypanosoma* sp. CC2016 B069
B082	Australian magpie (*Cracticus tibicen*)	T	*Trypanosoma* sp. AAT
B084	Australian magpie (*Cracticus tibicen*)	B	*Trypanosoma* sp. AAT
B091	Silver gull (*Chroicocephalus novaehollandiae*)	Sm	*Trypanosoma* sp. AAT

^a^Juvenile birds

### Ultrastructural analysis of *Trypanosoma* sp. AAT

**Fig. 2:**
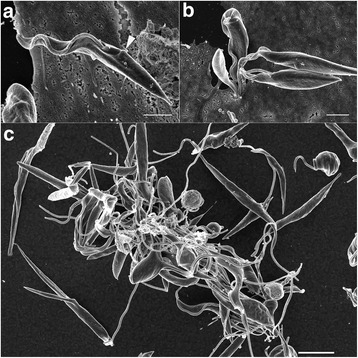
Scanning electron micrographs of *Trypanosoma* sp. AAT in culture. **a** Single small epimastigote. **b** Epimastigotes attached to outside of mammalian cells when grown at 37 °C for 24 h. **c** Epimastigotes grown at 27 °C in TRPMI dividing in nest (when trypanosomes divide while connected together). *Scale-bars*: **a**, 2 μm; **b**, 2 μm; **c**, 5 μm

**Fig. 3:**
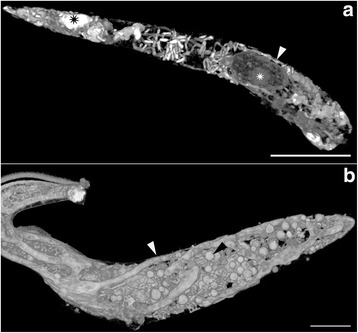
High resolution 3-dimentional electron microscopy images of volume rendering of *Trypanosoma* sp. AAT following FIB-SEM. **a** Larger epimastigote exhibiting fine structure of nucleus and nucleous (*white asterix*), reduced mitochondrion (*white arrow*), and reservosomes (*black asterix*). **b** Smaller epimastigote exhibiting mitochondrion (*white arrow*), and glycosomes (*black arrow*) (Additional file 2). *Scale-bars*: **a**, 5 μm; **b**, 2 μm

**Fig. 4:**
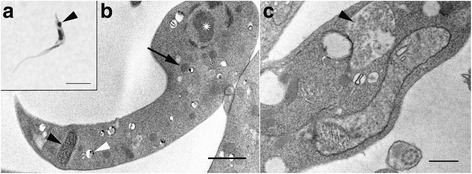
Trypomastigote of *Trypanosoma* sp. AAT. **a** Light micrograph exhibiting trypomastigote recognisable by posterior kinetoplast (*arrowhead*). **b** Transmission electron microscopy of trypomastigote exhibiting the kinetoplast (*black arrowhead*), nucleus (*asterisk*), acidocalcisomes (*white arrowhead*), and glycosomes (*black arrow*). **c** Reservosome in *Trypanosoma* sp. AAT (*arrow*). *Scale-bars*: **a**, 5 μm; **b**, **c**, 1 μm

**Fig. 5:**
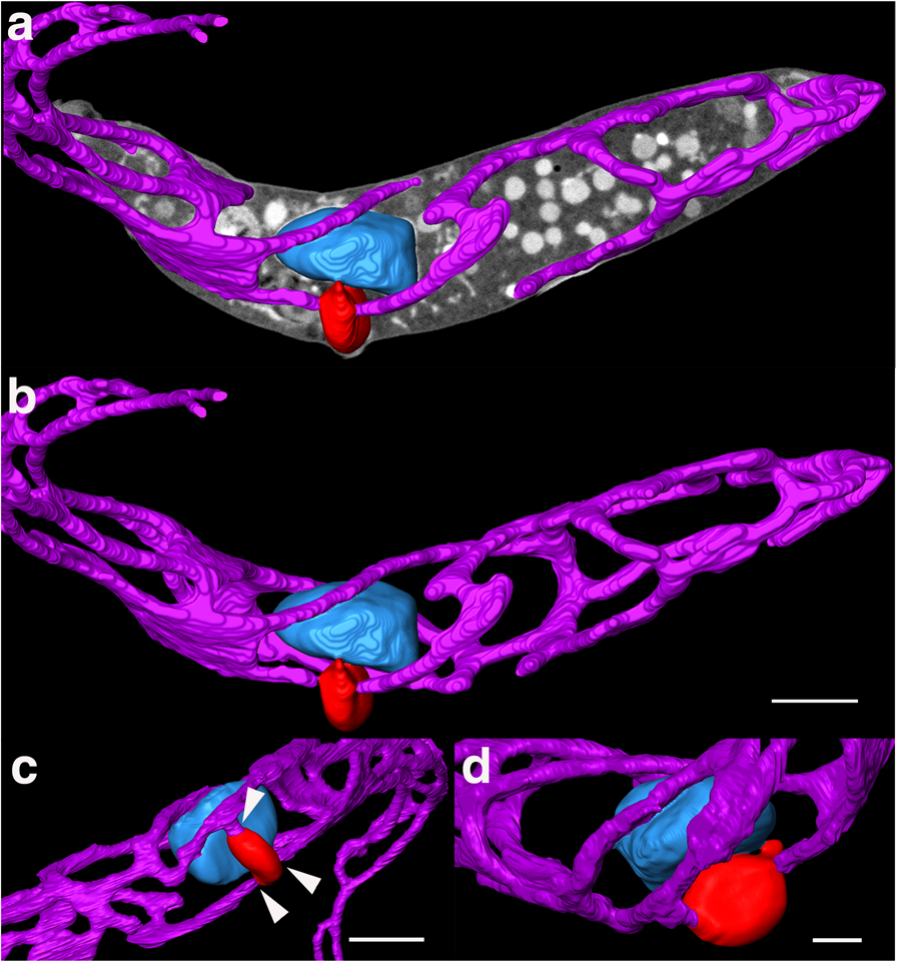
High resolution, 3-dimentional, FIB-SEM images of *Trypanosoma* sp. AAT small epimastigote DNA containing structures. Images were constructed using segmentation. **a** Segmented mitochondrion (*pink*), kinetoplast pocket containing kDNA (*red*), and nucleus (*blue*) shown with image collected from the electron beam to indicate the position of the organelles inside the cell. **b** 3D model of DNA containing structures: mitochondrion (*pink*), kinetoplast pocket (*red*), and nucleus (*blue*). **c** 3D model of kinetoplast pocket (*red*), which can be attached at multiple places to the mitochondrion (*white arrows*). **d** Round disc-shaped kinetoplast pocket (*red*) (Additional file 2). Scale-bars: **a**, **b**, 2 μm; **c**, 1 μm; **d**, 0.5 μm

**Fig. 6:**
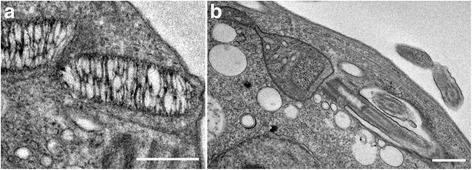
Transmission electron microscopy images of *Trypanosoma* sp. AAT kinetoplasts. **a** Elongated and loosely bound mini and maxi-circles within the boundaries of the kinetoplast. **b** The kinetoplast inside the mitochondrion-kinetoplast. *Scale-bars*: **a**, 0.5 μm; **b**, 1 μm



*Trypanosoma* sp. AAT grown in culture varied in size and appearance, although the majority were present as epimastigotes of two different sizes when measured along the cell axis from anterior to posterior (Fig. 2); a representative small individual 28 ± 3 μm (Fig. 2a) and a larger one 41 ± 4 μm (mean ± SD, *n* = 25) (Fig. 3a), are shown. Trypomastigotes were occasionally observed, recognisable due to the posterior placement of the kinetoplast in relation to the nucleus (Fig. 4a). The position of the kinetoplast is indicated by an arrow in the small epimastigote (Fig. 2a). When grown in the presence of cells, epimastigotes attached to the surface of the cells by their flagella, which. This has been noticed observed in other *Trypanosoma* spp., especially with epimastigotes within the gut of an invertebrate vector (Fig. 2b) [52–54]. The larger epimastigote can also be seen in 3D volumetric analysis exhibiting reservosomes (late endosomes) which are recognisable by their (i) irregular structure that contains folded monolayers and a mottled appearance, and (ii) their position, which is usually in the posterior region (Fig. 4b) [55]. The reservosomes in the large *Trypanosoma* sp. AAT epimastigotes are also present in the posterior region (Fig. 3a). Consistent with this, the larger epimastigotes also had a reduced mitochondrion and elongated glycosomes, compared to the small epimastigote, which had round glycosomes (Fig. 3b [56]. Acidocalcisomes are recognisable as black/white, round, organelles in TEM, which are used in several cell functions including storage of calcium (Fig. 4a) [57–59]. The smaller epimastigote also possessed reservosomes, acidocalicisomes, and glycosomes but these were rounder in shape (Fig. 3). The entire kinetoplast-mitochondrion structure isolated using segmentation from *Trypanosoma* sp. AAT small epimastigotes, revealed the branching cage-like structure of the single mitochondrion extending the length of the trypanosome and situated just under the subpellicular microtubules (Fig. 5a, b and Additional file 2). The kinetoplast pocket exhibited a disc-shape (Fig. 5d) close to the basal body to which they are connected *via* the TAC [24] and was positioned perpendicular to the mitochondrion in both small and large epimastigotes (Figs. 3 and 5c). The average length of the kDNA inside the kinetoplast pocket was 1.06 ± 0.10 μm and the average width or thickness was 0.39 ± 0.03 μm (mean ± SD, *n* = 25) (Fig. 6). Volumetric analysis of 25 trypanosomes with 1K1N (1 kinetoplast and 1 nucleus) gave an average kDNA volume of 0.33 ± 0.10 μm^3^ (mean ± SD, *n* = 25) and an average surface area of 2.80 ± 0.60 μm^2^ (mean ± SD, *n* = 25) (Additional file 3: Figure S1).

**Additional file 2:** High resolution 3D electron microscopy volume rendering of *Trypanosoma* sp. AAT small epimastigote. Images for the video were acquired using FIB-SEM, including individual sections, and segmented organelles; kinetoplast (*red*), nucleus (*blue*) and mitochondrion (*pink*). (MPG 48,541 kb)


### The universal minicircle sequence and binding protein in *Trypanosoma* sp. AAT

**Fig. 7:**
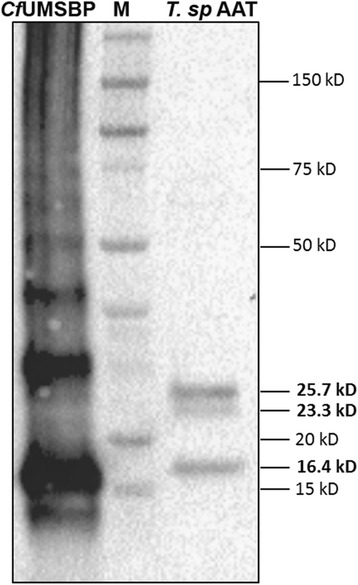
Western blot analysis using *Crithidia fasciculata* UMSBP antibodies. Total protein cell extracts from *Trypanosoma* sp AAT and *C. fasciculata* recombinant UMSBP were used. CfUMSBP: recombinant UMSBP from *C. fasciculata*. Apparent molecular masses (M) of standard proteins are indicated in kilodaltons (kD)

A 275 bp fragment of *Trypanosoma* sp. AAT minicircle was amplified (GenBank accession number KY498637) and aligned with the minicircles of *T. cruzi*, *T. rangeli*, and *T. copemani* from GenBank*.* The three conserved sequence blocks previously reported in all trypanosomatids were identified. The first (CBS-1:10 bp) and second blocks (CBS-2: 8 bp) of *Trypanosoma* sp. AAT, shared only 3 and 2 bases with the blocks of *T. cruzi, T. rangeli*, and *T. copemani* (Table 2)*.* The third CSB-3 block or UMS (12 bp) was identical to other *Trypanosoma* species (Table 2). *Crithidia fasciculata* anti-Universal Minicircle Binding Protein (UMSBP) antibodies recognised three peptides of approximately 16.4 kD, 23.3 kD, and 25.7 kD in *Trypanosoma* sp. AAT protein extracts (Fig. 7). When *C. fasciculata* recombinant UMSBP was used (positive control for hybridisation), a peptide of ~15.1 kD and a series of higher UMSBP oligomeric forms were recognised (Fig. 7).

**Table 2:** *Trypanosoma* sp. AAT kinetoplast minicircle conserved sequence blocks CSB-1, CSB-2, and CSB-3 (UMS). *Trypanosoma* sp. AAT and other *Trypanosoma* spp. are shown. Differences are shown in bold

Organism	CSB-1	CSB-2	CSB-3 or UMS	Reference
*T. rangeli*	AGGGGCGTTC	CCC-GTAC	GGGGTTGGTGTA	Barrois et al [74]
*T. cruzi* Y strain	AGGGGCGTTC	CCCCGTAC	GGGGTTGGTGTA	González et al. [75]
*T. copemani* G1	AGGGGCGTTC	CCCCGTAC	GGGGTTGGTGTA	Botero et al. [73]
*T. copemani* G2	AGGGGCGTTC	CCCCGTAC	GGGGTTGGTGTA	Botero et al. [73]
*Trypanosoma* sp. AAT	**GATAAG**GT**AG**	**ATGT**GT**TG**	GGGGTTGGTGTA	This study

## Discussion

### Cross-continental avian *Trypanosoma* spp.

Microscopy or culture of tissues from birds in the past did not yield any positive trypanosome identification [5], but in the present study many tissues tested positive by PCR. This demonstrates that tissues can be a valid tool for identifying *Trypanosoma* spp. infection. *Trypanosoma* spp. in Western Australia are diverse, cross-continental in distribution, and non-species specific, which is consistent with recent studies of avian trypanosomes elsewhere [8, 13, 26, 60]. Four *Trypanosoma* spp. isolated in Western Australia including *T. avium*, *T. thomasbancrofti, T. culicavium*, and an unknown *Trypanosoma* spp. (group C-111 in Zidková et al. [8]) have an intercontinental distribution. Of these, *T. avium* was the most common trypanosome found in eight of 18 infected birds. The most recently described avian *Trypanosoma* spp. is *T. thomasbancrofti* that was found in regent honeyeaters, and now in a boobook, Australian magpie, and a falcon. Before its formal description *T. thomasbancrofti* was found in a number of birds in Europe [8]. This is the first report of *T. culicavium* in Australia, which was identified in *Culex* mosquitos and the collared flycatcher (*Ficedula albicollis*) in central Europe [33]. A currently unnamed isolate from the group designated *Trypanosoma* spp. [13] and group C-III (Zidková et al. [8]) identified in birds and mosquitoes in central Europe was found for the first time in Australia, in an Australian mudlark. While most of the Australian birds infected with these *Trypanosoma* spp. are non-migratory outside Australia they often have wide distributions across Australia and into numerous pacific islands. However, the bird species infected with the *Trypanosoma* spp. identified are not closely related. For example, *T. avium* was found in seven different avian species that belong in four different orders including; Passeriformes (Australian magpie and raven), Strigiformes (boobook and barn owl), Falconiformes (falcon), and Columbiformes (pigeon, Australian dove), which raises questions about why avian trypanosome species are so conserved. A possible reason for the relatively conserved number of avian trypanosomes include a high level of vectorial specificity, thus involving invertebrate species that are very common and occur on all continents. However, at this stage little is known of the vectors in Australia [9, 61], while avian trypanosome vectors are considered to be biting flies or mosquitos elsewhere in the world [26, 33]. In Australia, leeches [14], biting flies [15], and ticks [62] have been suggested most frequently in the literature as vector candidates for avian trypanosomes.

### Australian avian *Trypanosoma* isolates

One novel isolate was identified in the study (*Trypanosoma* sp. CC2016 B002) from a boobook, which is a native owl widely distributed across Australia and a number of pacific islands. However, only a partial region of nuclear DNA was sequenced from a single sample, which is insufficient to characterise a new species. *Trypanosoma* sp. CC2016 B002 was positioned between *T. bennetti* and *T. culicavium* in phylogenetic analysis. A subsequent isolate found only in Australia is *Trypanosoma* sp. AAT, which was identified in a currawong as well as two Australian magpies, a silver gull (*Chroicocephalus novaehollandiae*), and a pink and grey galah (*Eolophus roseicapilla*). These birds are all native to Australia and occasionally migrate within its borders depending on temperature or food source availability [63] explaining the presence of *Trypanosoma* sp. AAT on both west (this study) and east [14] coasts of Australia. While *Trypanosoma* sp. AAT has only been found in Australia, it is closely related to *T. corvi* in both nuclear and mitochondrial gene regions [14], and genotypes from the *T. corvi* species clade have been identified in numerous bird species worldwide [8]. Of the common bird trypanosomes found worldwide the species not identified in the current study were *T. bennetti* and *T. corvi*, which is interesting as it has been suggested that most avian trypanosomes fall into one of three species, *T.avium, T. corvi* or *T. bennetti* [26]. The close genetic relationship observed between *Trypanosoma* sp. CC2016 B002/*T. thomasbancrofti* and *Trypanosoma* sp. AAT/*T. corvi* suggest larger studies incorporating more bird species and larger sample sizes may identify additional novel isolates, including those of *T. bennetti* and *T. corvi*.

Another trypanosome found in an avian host for the first time was *Trypanosoma* sp. CC2016 B069 isolated from an Australian raven. *Trypanosoma* sp. CC2016 B069 was highly similar to *Trypanosoma* sp. TL.AQ.22 in phylogenetic analysis and the region of 18S rDNA that was amplified in this study differing at 10 sites in a 494 bp fragment. *Trypanosoma* sp. TL.AQ.22 was previously reported from an Australian haemadipsid leech and placed in the *Trypanosoma theileri* clade [14]. The original study that isolated *Trypanosoma* sp. TL.AQ.22 found it was similar to *Trypanosoma cyclops* isolated from a monkey in Asia, *Trypanosoma* sp. ABF found in a wallaby, and a number of other isolates from terrestrial leaches [14]. This led the researchers to conclude leeches from the family Haemadipsidae could be important vectors in Australia and Asia. The present study is not the first to identify similar trypanosomes infecting a mammal and a bird. A partial sequence of *Trypanosoma* 18S rDNA that was obtained from a boodie (*Bettongia lesueur*) was identical to the avian *Trypanosoma* sp. AAT [64]. Both these organisms could be transmitted by the same vector, supporting the proposal that bird trypanosomes are similar due to high specificity towards the vector. However, birds and mammals being infected with the same trypanosome species is contradictory to the specificity of avian trypanosomes observed across continents. It is difficult to understand the path of transmission when so little is known about the life-cycles, vectors, and biology of avian and Australian *Trypanosma* spp.

### *Trypanosoma* sp. AAT ultrastructure

The majority of investigations into avian trypanosomes have involved analysis of trypomastigotes present in blood. Here, however the epimastigote stage was investigated because it is the morphological form that is grown in vitro and it has become increasingly clear that this is a more appropriate way to investigate their biology [6, 9]. With this, there are few *Trypanosoma* spp. to compare with the epimastigote biology of *Trypanosoma* sp. AAT. Despite their close genetic relationship and similar kinetoplast width, the description of *Trypanosoma* sp. AAT in vitro otherwise differs considerably when compared to the morphology of the closely related *T. corvi*. Three morphotypes were observed of *Trypanosoma* sp. AAT and both epimastigote forms were significantly larger than *T. corvi* epimastigotes [7, 33]. *T. corvi* had one epimastigote (19 ± 2 μm) when grown in culture [33], while a description from bone marrow identified a number of morphotypes including epimastigotes (shorter than 20 μm) with tapered ends, and trypomastigotes [7]. *Trypanosoma* sp. AAT kDNA appear most similar to *T. corvi* and *T. culicavium*, and were less similar to those of *T. avium* or *T. thomasbancrofti*, which are much stouter in TEM micrographs [13, 26, 33]. The kDNA width of *Trypanosoma* sp. AAT (kDNA width = 0.39 μm) did not differ from *T. corvi* (kDNA width = 0.39 μm), but was significantly different to *T. culicavium* (kDNA width = 0.31 μm) [26, 33]. However, there is considerable variation in kDNA width between individuals even when measuring them using volume analysis, which is more accurate than measuring cross-sections used for TEM analysis. The variation is present even though only 1K1N trypanosomes were counted in the analysis, which implies there is some variation in the size of the kinetoplast within the epimastigote stage. *Trypanosoma* sp. AAT is most likely a separate species from *T. corvi* based on morphology as well as differences in 18S rDNA and *GAPDH* gene regions that were observed [14]. Although, the limited structural and molecular information available on avian trypanosomes combined with a high level of diversity within apparent species groups has resulted in unclear species boundaries between isolates. At this stage more information on avian trypanosomes is required before *Trypanosoma* sp. AAT can be conclusively placed within *T. corvi* or characterised as a new species.

The first comprehensive high-resolution, 3-dimensional, ultrastructural study of *Trypanosoma* sp. AAT revealed that the kinetoplast is disc-shaped and perpendicular to the rest of the mitochondrion. The mitochondrion was observed under the subpellicular microtubules in the smaller epimastigotes and is a vast branching structure occupying a large volume in the cytoplasm. In *T. cruzi* and *T. dionisii* 3D reconstructions, the epimastigote mitochondrion is extensive and positioned in the same place as *Trypanosoma* sp. AAT - just under the subpellicular microtubules -, the kinetoplast pocket is positioned underneath the nucleus not alongside it [39, 65]. In *T. cruzi* amastigotes 3D reconstructions revealed the mitochondrion has a horse shoe shape [18, 66] while *T. brucei* trypomastigotes in the vertebrate host have a poorly developed branching mitochondrion [17, 35, 36]. Direct glycolysis may be used in the presence of high glucose concentrations (such as the blood) reducing the size of the mitochondrion between morphological forms and increasing the size of the glycosomes, which was observed in *Herpetomonas roitmani* [67] and *T. brucei* [20, 21] suggesting the parasites switch from oxidative phosphorylation to glycolysis. This process may reverse in the presence of low glucose environments such as the invertebrate gut resulting in a larger mitochondrion [22, 68]. A smaller reduced mitochondrion was observed in the larger epimastigotes compared to the smaller epimastigotes grown in the same medium and harvested at the same time in this study. Therefore, differences in size between epimastigote forms was not solely due to nutrient availability, but could be cell-cycle dependent or due to differences in how various morphological forms metabolise and develop. Epimastigotes occur in the bone marrow of birds [7] as well as the invertebrate vector, while the blood contains trypomastigotes as in other *Trypanosoma* spp. [33, 69]. This is interesting as it could be hypothesised that the larger *Trypanosoma* sp. AAT epimastigote is likely to occur in bone marrow where there is a high glucose content and the smaller epimastigote in the insect vector that is likely more reliant on L-proline for a carbon source [22]. The size and shape of the reservosomes, glycosomes, and acidocalcisomes also differs between the two sizes of epimastigotes observed in *Trypanosoma* sp. AAT that is both cell cycle and nutrient dependent in other trypanosomes, especially *T. brucei*, which has been extensively studied [18, 19, 56, 68]. Put simply, due to the similarity in nutrient availability for the two epimastigotes observed in this study, differences in organelle structure is most likely cell-cycle dependent.

Parasitism is likely to have emerged in trypanosomatids due to this group of organisms containing both free-living and obligate parasites, and the shape and structure of the kinetoplast is important in understanding the evolution of trypanosomes [70, 71]. The kDNA is simply dispersed throughout the mitochondrial matrix in early trypanosomatids such as bodonids, while in avian trypanosomes the minicircles are larger than that of the later branching trypanosomes including *T. brucei* and *T. cruzi* [31]. Two of the three minicircle sequence blocks common amongst late branching trypanosomes like *T. cruzi* (CSB-1 and CSB-2) are quite different compared to those of *Trypanosoma* sp. AAT. Avian trypanosomes have larger minicircles compared to other trypanosomes, so the differences in these regions are not unexpected [31]. However, there are aspects of kDNA that are highly conserved. The 12-mer UMS (or CSB-3), which is present in the minicircles of all trypanosomatids investigated [27], was found in *Trypanosoma* sp. AAT. The UMS binding protein that is involved in kDNA replication, which is found in other kinetoplastids such as *C. fasciculata*, *T. cruzi*, *L. donovani* and *T. brucei* [28–30, 72], was also present in *Trypanosoma* sp. AAT. Although, results suggested *Trypanosoma* sp. AAT contains three different UMSBPs. Interestingly, the size of two *Trypanosoma* sp. AAT UMSBPs (16.4 and 25.7 kD) was exactly the same as the UMSBPs found on the minicircles of the Australian marsupial trypanosome *T. copemani* [73].

## Conclusions

The molecular and structural information provided in this study contribute to understanding the diversity and structural biology of avian trypanosomes. While it is clear avian trypanosomes are cross-continental and exist in fewer species groups than are currently described, further studies incorporating molecular and structural techniques are required to establish the true extent of bird trypanosome diversity. Despite the close relationship with *T. corvi*, *Trypanosoma* sp. AAT is most likely a separate species currently found only in Australia. However, more information is required on avian trypanosomes in general, in order to provide clearer species boundaries. The first 3-dimentional ultrastructural analysis of an avian trypanosome provides interesting information on their morphology and organelle arrangement. Although, a greater understanding of avian trypanosome ultrastructure and metabolism, would assist in understanding their life-cycles and the impact of infection on their hosts. The possibility that avian *Trypanosoma* spp. are also infecting mammals and could be transmitted by haemadipsid leeches should be further investigated. Additional research on the trypanosomes infecting birds and their vectors will hopefully explain why so many avian trypanosomes are cross-continental but not species-specific.

## Additional files

Additional file 1: Table S1. Tissue samples collected from 93 birds. Bird identification number (ID), bird species, and tissues extracted from each individual are included. Tissues extracted include heart (H), liver (L), spleen (S), kidney (K), lung (Ln), striated or skeletal muscle (Sm), brain (B), and thoracic muscle (T). (DOCX 31 kb)

Additional file 3: Figure S1. Kinetoplasts (*n* = 11) extracted from dataset of serially sectioned *Trypanosoma* sp. AAT. **a** Whole dataset collected using FIB-SEM. **b** A single slice collected using FIB-SEM. **c** The final slice from the dataset including all the extracted kinetoplasts from the whole dataset in different colours. **d** Graphs exhibiting variation between individual kinetoplasts examined in volume analysis including volume, surface area, length and width. *Scale-bars*: 2 μm. (TIF 5108 kb)

## References

[1] Baker JR (1956). Studies on *Trypanosoma avium* Danilewsky 1885 I. Incidence in some birds of Hertfordshire. Parasitology.

[2] Baker JR (1956). Studies on *Trypanosoma avium* Danilewsky 1885 III. Life cycle in vertebrate and invertebrate hosts. Parasitology.

[3] Stabler RM, Holt PA, Kitzmiller NJ (1966). *Trypanosoma avium* in the blood and bone marrow from 677 Colorado birds. J Parasitol.

[4] Baker JR (1974). Protozoan parasites of the blood of British wild birds and mammals. J Zool Lond.

[5] Apanis V (1991). Avian trypanosomes as models of hemoflagellate evolution. Parasitol Today.

[6] Votýpka J, d'Avila-Levy CM, Grellier P, Maslov DA, Lukeš J, Yurchenko V (2015). New approaches to systematics of Trypanosomatidae: criteria for taxonomic (re)description. Trends Parasitol.

[7] Nandi NC, Bennett F (1994). Rediscription of *T. corvi* Stephans & Christophers, 1908, emend. Baker 1976 and remarks on the trypanosomes of the avian family Corvidae. Mem Inst Oswaldo Cruz.

[8] Zídková L, Cepicka I, Szabová J, Svobodová M (2012). Biodiversity of avian trypanosomes. Infect Genet Evol.

[9] Cooper C, Clode PL, Peacock C, Thompson RCA. Host-parasite relationships and life histories of trypanosomes in Australia. Adv Parasitol. 2016. (In press). 10.1016/bs.apar.2016.06.001 28325373

[10] Mackerras MJ, Mackerras IM (1959). The haematozoa of Australian birds. Aust J Zool.

[11] Cleland JB, Johnston TH (1910). The haematozoa of Australian birds. No. 1.. Trans Roy Soc South Aust.

[12] Cleland JB, Johnston TH (1911). The haematozoa of Australian birds. No. 2.. Trans Roy Soc South Aust.

[13] Šlapeta J, Morin-Adeline V, Thompson P, Mcdonell D, Shiels M, Gilchrist K (2016). Intercontinental distribution of a new trypanosome species from Australian endemic Regent Honeyeater (*Anthochaera phrygia*). Parasitology.

[14] Hamilton PB, Gidley J, Stevens JR, Holz P, Gibson WC (2005). A new lineage of trypanosomes from Australian vertebrates and terrestrial bloodsucking leeches (Haemadipsidae). Int J Parasitol.

[15] Botero A, Cooper C, Thompson CK, Clode PL, Rose K, Thompson RCA (2016). Morphological and phylogenetic description of *Trypanosoma noyesi* sp. nov.: A Australian wildlife trypanosome within the *T. cruzi* clade. Protist.

[16] Simpson L (1973). Structure and function of kinetoplast DNA. J Protozool.

[17] Jakob M, Hoffmann A, Amodeo S, Peitsch C, Zuber B, Ochsenreiter T (2017). Mitochondrial growth during the cell cycle of *Trypanosoma brucei* bloodstream forms. Sci Rep.

[18] De Souza W, Attias M, Rodrigues CF (2009). Particularities of mitochondrial structure in parasitic protists (Apicomplexa and Kinetoplastida). Int J Biochem Cell Biol.

[19] Martins AV, Gomes AP, Gomes de Mendonça E, Rangel Fietto JL, Santana LA, de Almeida Oliveira MG (2012). Biology of *Trypanosoma cruzi*: an update. Infection.

[20] Böhringer S, Hecker H (1974). Quantitative ultrastructural differences between strains of the *Trypanosoma brucei* subgroup during transformation in blood. J Protozool.

[21] Böhringer S, Hecker H (1975). Quantitative ultrastructural investigations of the lifecycle of *Trypanosoma brucei*: a morphometric analysis. J Protozool.

[22] Bringaud F, Rivière L, Coustou V (2006). Energy metabolism of trypanosomatids: adaptation to available carbon sources. Mol Biochem Parasitol.

[23] Dias JCP, Macedo VO, Coura JR (2005). Doenca de Chagas. Dinâmica das Doenças Infecciosas e Parasitárias.

[24] Ogbadoyi EO, Robinson DR, Gull K (2003). A high-order trans-membrane structural linkage is responsible for mitochondrial genome positioning and segregation by flagellar basal bodies in trypanosomes. Mol Biol Cell.

[25] Hajduk S, Ochsenreiter T (2010). RNA editing in kinetoplastids. RNA Biol.

[26] Votýpka J, Lukeš J, Oborník M (2004). Phylogenetic relationship of *Trypanosoma corvi* with other avian trypanosomes. Acta Protozool.

[27] Ray DS (1989). Conserved sequence blocks in kinetoplast minicircles from diverse species of trypanosomes. Mol Cell Biol.

[28] Milman N, Motyka SA, Englund PT, Robinson D, Shlomai J (2007). Mitochondrial origin-binding protein UMSBP mediates DNA replication and segregation in trypanosomes. Proc Natl Acad Sci USA.

[29] Coelho ER, Ürményi TP, Franco da Silveira J, Rondinelli E, Silva R (2003). Identification of PDZ5, a candidate universal minicircle sequence binding protein of *Trypanosoma cruzi*. Int J Parasitol.

[30] Singh R, Purkait B, Abhishek K, Saini S, Das S, Verma S (2016). Universal minicircle sequence binding protein of *Leishmania donovani* regulates pathogenicity by controlling expression of cytochrome-b. Cell Biosci.

[31] Yurchenko V, Hobza R, Benada O, Lukeš J (1999). *Trypanosoma avium*: Large minicircles in the kinetoplast DNA. Exp Parasitol.

[32] Lukeš J, Yurchenko V (2000). *Trypanosoma avium*: Novel features of the kinetoplast structure. Exp Parasitol.

[33] Votýpka J, Szabová J, Rá drova J, Zidková L, Svobodová M (2012). *Trypanosoma culicavium* sp. nov., an avian trypanosome transmitted by *Culex* mosquitoes. Int J Syst Evol Microbiol.

[34] Lacomble S, Vaughan S, Gadelha C, Morphew MK, Shaw MK, McIntosh JR, Gull K (2010). Basal body movements orchestrate membrane organelle division and cell morphogenesis in *Trypanosoma brucei*. J Cell Sci.

[35] Vanwalleghem G, Fontaine F, Lecordier L, Tebabi P, Klewe K, Nolan DP (2015). Coupling of lysosomal and mitochondrial membrane permeabilization in trypanolysis by APOL1. Nat Commun.

[36] Hughes L, Borrett S, Towers K, Starborg T, Vaughan S (2017). Patterns of organelle ontogeny through a cell cycle revealed by whole-cell reconstructions using 3D electron microscopy. J Cell Sci.

[37] Girard-Dias W, Alcântara CL, Cunha-e-Silva N, De Souza W, Miranda K (2012). On the ultrastructural organization of *Trypanosoma cruzi* using cryo-preparation methods and electron tomography. Histochem Cell Biol.

[38] Alcantara CL, Vidal JC, De Souza W, Cunha-e-Silva NL (2014). The three-dimensional structure of the cytostome-cytopharynx complex of *Trypanosoma cruzi* epimastigotes. J Cell Sci.

[39] Oliveira MP, Ramos TCP, Pinheiro AMVN, Bertini S, Takahashi HK, Straus AH, Haapalainen EF (2013). Tridimensional ultrastructure and glycolipid pattern studies of *Trypanosoma dionisii*. Acta Trop.

[40] Deerinck TJ, Bushong EA, Lev-Ram V, Shu X, Tsien RY, Ellisman MH (2010). Enhancing serial block-face scanning electron microscopy to enable high resolution 3-D nanohistology of cells and tissues. Microscop Microanal.

[41] Maslov DA, Lukeš J, Jirku M, Simpson L (1996). Phylogeny of trypanosomes as inferred from the small and large subunit rRNAs: implications for the evolution of parasitism in the trypanosomatid protozoa. Mol Biochem Parasitol.

[42] McInnes LM, Gillett A, Ryan UM, Austen J, Campbell RS, Hanger J, Reid SA (2009). *Trypanosoma irwini* n. sp. (Sarcomastigophora: Trypanosomatidae) from the koala (*Phascolarctos cinereus*). Parasitology.

[43] Noyes HA, Stevens JR, Teixeira M, Phelan J, Holz P (1999). A nested PCR for the ssrRNA gene detects *Trypanosoma binneyi* in the platypus and *Trypanosoma* sp. in wombats and kangaroos in Australia. Int J Parasitol.

[44] Tautz D, Renz M (1983). An optimized freeze-squeeze method for the recovery of DNA fragments from agarose gels. Anal Biochem.

[45] Kearse M, Moir R, Wilson A, Stones-Havas S, Cheung M, Sturrock S (2012). Geneious Basic: an integrated and extendable desktop software platform for the organization and analysis of sequence data. Bioinformatics.

[46] Edgar RC (2004). MUSCLE: multiple sequence alignment with high accuracy and high throughput. Nucleic Acids Res.

[47] Posada D (2008). jModelTest: phylogenetic model averaging. Mol Biol Evol.

[48] Tamura K, Stecher G, Peterson D, Filipski A, Kumar S (2013). MEGA6: molecular evolutionary genetics analysis version 6.0. Mol Biol Evol.

[49] Ronquist F, Huelsenbeck JP (2003). MrBayes 3: Bayesian phylogenetic inference under mixed models. Bioinformatics.

[50] Grace TDC, Brzostowski HW (1966). Analysis of the amino acids and sugars in an insect cell culture medium during cell growth. J Insect Physiol.

[51] Kizilyaprak C, Longo G, Daraspe J, Humbel BM (2015). Investigation of resins suitable for the preparation of biological sample for 3-D electron microscopy. J Struct Biol.

[52] Hoare CA (1972). The trypanosomes of mammals. A zoological monograph.

[53] Vickerman K (1973). The mode of attachment of *Trypanosoma vivax* in the proboscis of the tsetse fly *Glossina fuscipes*: an ultrastructural study of the epimastigote stage of the trypanosome. J Protozool.

[54] Van den Abbeele J, Claes YC, Van Bockstaele D, Le Ray D, Coosemans M (1999). *Trypanosoma brucei* spp. development in the tsetse fly: characterisation of the post-mesocyclic stages in the foregut and proboscis. Parasitology.

[55] Sant'Anna C, Pereira MG, Lemgruber L, de Souza W, Cunha e Silva NL. New insights into the morphology of *Trypanosoma cruzi* reservosome. Microsc Res Tech. 2008;71:599–605. 10.1002/jemt.20592 18452191

[56] De Souza W (2008). Electron microscopy of trypanosomes - a historical view. Mem Inst Oswaldo Cruz.

[57] Miranda K, Docampo R, Benchimol M, de Souza W (2000). The fine structure of acidocalcisomes in *Trypanosoma cruzi*. Parasitol Res.

[58] Miranda K, Docampo R, Grillo O, de Souza W (2004). Acidocalcisomes of trypanosomatids have species specific elemental composition. Protist.

[59] Docampo R, de Souza W, Miranda K, Rohloff P, Moreno SNJ (2005). Acidocalcisomes - conserved from bacteria to man. Nat Rev Microbiol.

[60] Bennett GF (1961). On the specificity and transmission of some avian trypanosomes. Can J Zool.

[61] Thompson CK, Thompson RCA (2015). Trypanosomes of Australian mammals: knowledge gaps regarding transmission and biosecurity. Trends Parasitol.

[62] Austen JM, Ryan UM, Friend JA, Ditcham WGF, Reid SA (2011). Vector of *Trypanosoma copemani* identified as *Ixodes* sp. Parasitology.

[63] Christidis L, Boles WE (2008). Systematics and Taxonomy of Australian Birds.

[64] Averis S, Thompson RCA, Lymbery AJ, Wayne AF, Morris KD, Smith A (2009). The diversity, distribution and host-parasite associations of trypanosomes in Western Australian wildlife. Parasitology.

[65] Ramos TC, Haapalainen EF, Schenkman S (2011). Three-dimensional reconstruction of *Trypanosoma cruzi* epimastigotes and organelle distribution along the cell division cycle. Cytometry A.

[66] Newberry LB, Paulin JJ (1989). Reconstruction of the chondriome of the amastigote form of *Trypanosoma cruzi*. J Parasitol.

[67] Faria-e-Silva PM, Attias M, De Souza W (2000). Biochemical and ultrastructural changes in *Herpetomonas roitmani* related to the energy metabolism. Biol Cell.

[68] Michels PAM, Bringaud F, Herman M, Hannaert V (2006). Metabolic functions of glycosomes in trypanosomatids. Biochim Biophys Acta.

[69] Salakij C, Kasorndorkbua C, Lertwatcharasarakul P, Salakij J (2012). Hematology, molecular phylogeny and ultra-structure of *Trypanosoma corvi* in a Shikra. Comp Clin Pathol.

[70] Lukeš J, Lys Guilbride D, Votýpka J, Zíkova´ A, Benne R, Englund PT (2002). Kinetoplast DNA network: evolution of an improbable structure. Eukaryot Cell.

[71] Lukeš J, Skalický T, Týč J, Votýpka J, Yurchenkoa V (2014). Evolution of parasitism in kinetoplastid flagellates. Mol Biochem Parasitol.

[72] Tzfati Y, Abeliovich H, Kapeller I, Shlomai J (1992). A single-stranded DNA-binding protein from *Crithidia fasciculata* recognises the nucleotide sequence at the origin of replication of kinetoplast DNA minicircles. Proc Natl Acad Sci U S A.

[73] Botero A, Peacock C, Clode PL, Thompson RCA. Trypanosomes genetic diversity, polyparasitism and the population decline of the critically endangered Australian marsupial, the brush-tailed bettong or woylie (*Bettongia penicillata*). PhD thesis, Murdoch University; 2014. 10.1016/j.ijppaw.2013.03.001 PMC3862532 24533319

[74] Barrois M, Riou G, Galibert F. Complete nucleotide sequence of minicircle kinetoplast DNA from *Trypanosoma equiperdum*. Proc Natl Acad Sci USA. 1981;78(6):3323–7. 10.1073/pnas.78.6.3323 PMC319560 6267582

[75] González A (1986). Nucleotide sequence of a *Trypanosoma cruzi* minicircle. Nucl Acids Res.

