# Supplementary material for: A comparative molecular and 3-dimensional structural investigation into cross-continental and novel avian Trypanosoma spp. in Australia
Source: Parasit Vectors. 2017 May 12;10:234. doi: 10.1186/s13071-017-2173-x (PMC5427604; doi:10.1186/s13071-017-2173-x)
Supplement: Supplementary file 2 — Tissue samples collected from 93 birds. Bird identification number (ID), bird species, and tissues extracted from each individual are included. Tissues extracted include heart (H), liver (L), spleen (S), kidney (K), lung (Ln), striated or skeletal muscle (Sm), brain (B), and thoracic muscle (T). (DOCX 31 kb) [file 13071_2017_2173_MOESM1_ESM.docx]

**Additional File 1. Table S1.** Tissue samples collected from 93 birds. Bird identification number (ID), bird species, and tissues extracted from each individual are included. Tissues extracted include heart (H), liver (L), spleen (S), kidney (K), lung (Ln), striated or skeletal muscle (Sm), brain (B), and thoracic muscle (T).

| ***ID*** | ***Bird species*** | ***H*** | ***L*** | ***S*** | ***K*** | ***Ln*** | ***Sm*** | ***B*** | ***T*** |
| --- | --- | --- | --- | --- | --- | --- | --- | --- | --- |
| B001 | Pink and grey galah (*Eolophus roseicapilla*) | x | x | x | x | x | x | x |  |
| B002 | Boobook (*Ninox novaeseelandiae*) | x | x | x |  |  | x |  |  |
| B003 | Currawong^a^ (*Strepera* spp*.*) | x | x |  | x | x | x | x |  |
| B004 | Falcon (*Falco* spp*.*) | x | x | x | x | x | x | x |  |
| B005 | Corella (*Cacatua* spp.) | x | x | x | x | x | x | x |  |
| B006 | Pink and grey galah (*Eolophus roseicapilla*) | x | x | x | x | x | x | x |  |
| B007 | Pacific black duck (*Anas superciliosa*) | x | x | x | x | x | x | x |  |
| B008 | Barn owl (*Tyto alba*) | x | x |  | x | x | x | x |  |
| B009 | Kite (Family Accipitridae) | x | x | x | x | x | x | x |  |
| B010 | Kookaburra (*Dacelo novaeguineae*) | x | x | x | x | x | x | x |  |
| B011 | Australian dove^a^ (Family Columbidae) | x | x | x | x | x | x | x |  |
| B012 | Rainbow lorikeet^a^ (*Trichoglossus moluccanus*) | x | x | x | x | x | x | x |  |
| B013 | Rainbow lorikeet (*Trichoglossus moluccanus*) | x | x | x | x | x | x | x |  |
| B014 | Australian dove (Family Columbidae) | x | x | x | x | x | x | x |  |
| B015 | Singing honeyeater (*Gavicalis virescens*) | x | x |  | x | x | x | x |  |
| B016 | Australian dove^a^ (Family Columbidae) | x | x | x | x | x | x | x |  |
| B017 | Sacred kingfisher (*Todiramphus sanctus*) | x | x | x | x | x | x | x |  |
| B018 | Australian magpie (*Cracticus tibicen*) | x | x | x | x | x | x | x |  |
| B019 | Australian magpie^a^ (*Cracticus tibicen*) | x | x | x | x | x | x | x |  |
| B020 | Australian magpie (*Cracticus tibicen*) | x | x | x | x | x | x | x |  |
| B021 | Singing honeyater^a^ (*Gavicalis virescens*) | x | x | x | x | x | x | x |  |
| B022 | Kookaburra (*Dacelo novaeguineae*) | x | x | x | x | x | x | x |  |
| B023 | Australian magpie (*Cracticus tibicen*) | x | x | x | x | x | x | x |  |
| B024 | Boobook (*Ninox novaeseelandiae*) | x | x | x | x | x | x | x |  |
| B025 | Corella (*Cacatua* spp.) | x | x | x | x | x | x | x |  |
| B026 | Pink and grey galah (*Eolophus roseicapilla*) | x | x | x | x | x | x | x |  |
| B027 | Wilson's storm petrel (*Oceanites oceanicus*) | x | x | x | x | x | x | x |  |
| B028 | Rainbow lorikeet^a^ (*Trichoglossus moluccanus*) | x | x | x | x | x | x | x |  |
| B029 | Australian magpie^a^ (*Cracticus tibicen*) | x | x | x | x | x | x | x |  |
| B030 | Pigeon (*Columba livia*) | x | x | x | x | x | x | x |  |
| B031 | Pigeon (*Columba livia*) | x | x | x | x | x | x | x |  |
| B032 | Brown honeyeater (*Lichmera indistincta*) | x | x |  | x | x | x | x |  |
| B033 | New Holland Honeyeater (*Phylidonyris novaehollandiae*) | x | x | x | x | x | x | x |  |
| B034 | Barn owl (*Tyto alba*) | x | x | x | x | x | x | x |  |
| B035 | Falcon (*Falco* spp*.*) | x | x |  | x | x | x | x |  |
| B036 | Barn owl (*Tyto alba*) | x | x | x | x | x | x | x |  |
| B037 | Silvereye (*Zosterops lateralis*) | x | x | x | x | x | x | x |  |
| B038 | Australian mudlark (*Grallina cyanoleuca*) | x | x | x | x | x | x | x |  |
| B039 | Pink and grey galah (*Eolophus roseicapilla*) | x | x | x | x | x | x | x |  |
| B040 | Corella (*Cacatua* spp.) | x | x | x | x | x | x | x |  |
| B041 | Black cormorant (Phalacrocorax carbo) | x | x | x | x | x | x | x |  |
| B042 | Rainbow lorikeet (*Trichoglossus moluccanus*) | x | x | x | x | x | x | x |  |
| B043 | Pink and grey galah (*Eolophus roseicapilla*) | x | x | x | x | x | x | x |  |
| B044 | Pink and grey galah (*Eolophus roseicapilla*) | x | x | x | x | x | x | x |  |
| B045 | Frogmouth (*Podargus strigoides*) | x | x | x | x | x | x | x |  |
| B046 | Rainbow lorikeet (*Trichoglossus moluccanus*) | x | x | x | x | x | x | x |  |
| B047 | Corella (*Cacatua* spp.) | x | x | x | x | x | x | x |  |
| B048 | Pigeon 1660 leg band (*Columba livia*) | x | x | x | x | x | x | x |  |
| B049 | Brown honeyeater (*Lichmera indistincta*) | x | x |  | x | x | x | x |  |
| B050 | Australian raven (*Corvus coronoides*) |  |  |  |  |  |  | x | x |
| B051 | Australian raven (*Corvus coronoides*) |  |  |  |  |  | x |  |  |
| B052 | Australian raven (*Corvus coronoides*) |  |  |  |  |  | x | x | x |
| B053 | Australian raven (*Corvus coronoides*) |  |  |  |  |  | x | x |  |
| B054 | Australian raven (*Corvus coronoides*) |  |  |  |  |  | x | x | x |
| B055 | Australian raven (*Corvus coronoides*) |  |  |  |  |  | x |  | x |
| B056 | Australian raven (*Corvus coronoides*) |  |  |  |  |  | x | x | x |
| B057 | Australian raven (*Corvus coronoides*) |  |  |  |  |  | x |  | x |
| B058 | Australian raven (*Corvus coronoides*) |  |  |  |  |  | x | x | x |
| B059 | Australian raven (*Corvus coronoides*) |  |  |  |  |  | x | x | x |
| B060 | Australian raven (*Corvus coronoides*) |  |  |  |  |  | x |  |  |
| B061 | Australian raven (*Corvus coronoides*) |  |  |  |  |  | x | x | x |
| B062 | Australian raven (*Corvus coronoides*) |  |  |  |  |  | x |  |  |
| B063 | Australian raven (*Corvus coronoides*) |  |  |  |  |  | x | x | x |
| B064 | Australian raven (*Corvus coronoides*) |  |  |  |  |  | x |  | x |
| B065 | Australian raven (*Corvus coronoides*) |  |  |  |  |  |  | x |  |
| B066 | Australian raven (*Corvus coronoides*) |  |  |  |  |  | x | x | x |
| B067 | Australian raven (*Corvus coronoides*) |  |  |  |  |  | x | x |  |
| B068 | Australian raven (*Corvus coronoides*) |  |  |  |  |  | x | x | x |
| B069 | Australian raven (*Corvus coronoides*) |  |  |  |  |  | x | x | x |
| B070 | Australian raven (*Corvus coronoides*) |  |  |  |  |  | x | x | x |
| B071 | Australian raven (*Corvus coronoides*) |  |  |  |  |  | x |  |  |
| B072 | Australian raven (*Corvus coronoides*) |  |  |  |  |  | x | x | x |
| B073 | Australian raven (*Corvus coronoides*) |  |  |  |  |  | x | x | x |
| B074 | Australian raven (*Corvus coronoides*) |  |  |  |  |  |  | x | x |
| B075 | Australian raven (*Corvus coronoides*) |  |  |  |  |  | x | x | x |
| B076 | Australian raven (*Corvus coronoides*) |  |  |  |  |  |  | x |  |
| B077 | Australian raven (*Corvus coronoides*) |  |  |  |  |  |  | x |  |
| B078 | Australian raven (*Corvus coronoides*) |  |  |  |  |  |  | x |  |
| B079 | Australian raven (*Corvus coronoides*) |  |  |  |  |  |  |  | x |
| B080 | Australian magpie (*Cracticus tibicen*) |  |  |  |  |  |  | x |  |
| B081 | Australian magpie (*Cracticus tibicen*) |  |  |  |  |  | x | x | x |
| B082 | Australian magpie (*Cracticus tibicen*) |  |  |  |  |  | x | x | x |
| B083 | Australian magpie (*Cracticus tibicen*)roelands |  |  |  |  |  |  | x | x |
| B084 | Australian magpie (*Cracticus tibicen*) |  |  |  |  |  | x | x | x |
| B085 | Australian magpie (*Cracticus tibicen*)roelands |  |  |  |  |  | x | x |  |
| B086 | Australian magpie (*Cracticus tibicen*)roelands |  |  |  |  |  | x | x | x |
| B087 | Australian magpie (*Cracticus tibicen*) |  |  |  |  |  | x |  |  |
| B088 | Pacific black duck (*Anas superciliosa*) |  |  |  |  |  | x | x | x |
| B089 | Kookaburra (*Dacelo novaeguineae*) |  |  |  |  |  | x | x | x |
| B090 | Purple swamphen (*Porphyrio melanotus*) |  |  |  |  |  | x | x | x |
| B091 | Silver gull (*Chroicocephalus novaehollandiae*) |  |  |  |  |  | x |  |  |
| B092 | Silver gull (*Chroicocephalus novaehollandiae*) |  |  |  |  |  | x |  |  |
| B093 | Silver gull (*Chroicocephalus novaehollandiae*) |  |  |  |  |  |  | x |  |

^a^Juvenile birds
