# Supplementary figures and images for: A comparative molecular and 3-dimensional structural investigation into cross-continental and novel avian Trypanosoma spp. in Australia
Source: Parasit Vectors. 2017 May 12;10:234. doi: 10.1186/s13071-017-2173-x (PMC5427604; doi:10.1186/s13071-017-2173-x)

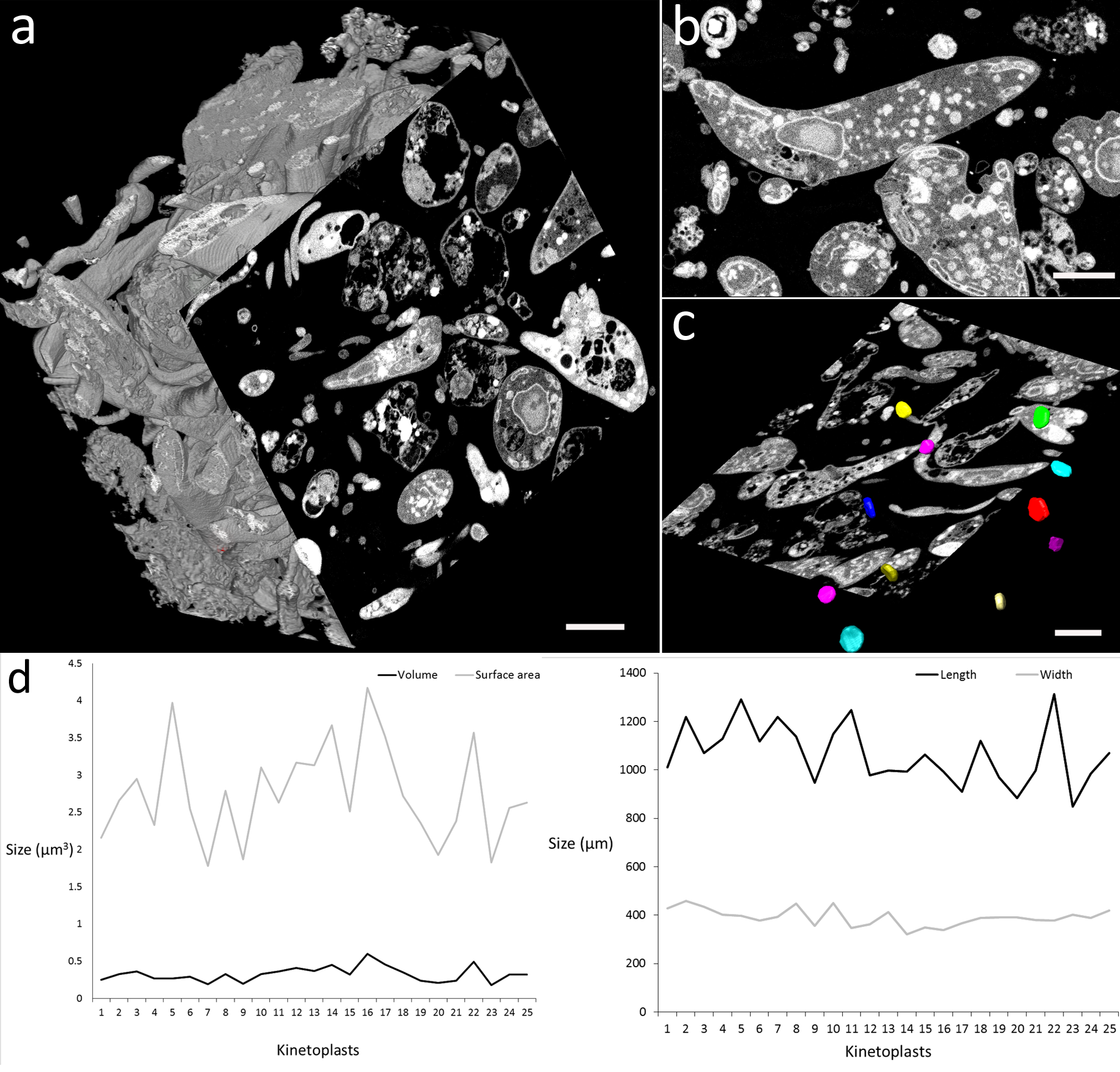

Supplement: Supplementary file 3 — Kinetoplasts (n = 11) extracted from dataset of serially sectioned Trypanosoma sp. AAT. a Whole dataset collected using FIB-SEM. b A single slice collected using FIB-SEM. c The final slice from the dataset including all the extracted kinetoplasts from the whole dataset in different colours. d Graphs exhibiting variation between individual kinetoplasts examined in volume analysis including volume, surface area, length and width. Scale-bars: 2 μm. (TIF 5108 kb) [file 13071_2017_2173_MOESM3_ESM.tif]
